# Physical exercise/melatonin interaction in young rats fed a low-protein diet: a behavioral, electrophysiological, and redox balance analysis

**DOI:** 10.3389/fnagi.2026.1740062

**Published:** 2026-02-06

**Authors:** Maria Luísa Figueira de Oliveira, Jennyfer Martins de Carvalho, José Anderson da Silva Gomes, Valéria Bianca de Souza Santos, Leucio Duarte Vieira Filho, Arthur Gabriel Alves Furtado de Carvalho Noya, Rubem Carlos Araujo Guedes

**Affiliations:** 1 Department of Physiology and Pharmacology, Biosciences Center, Federal University of Pernambuco, Recife, Brazil; 2 Department of Nutrition, Federal University of Pernambuco, Recife, Brazil

**Keywords:** anxiety, cortical spreading depression, malnutrition, melatonin, neuroplasticity, physical activity, redox imbalance

## Abstract

**Introduction:**

Early-life protein malnutrition disrupts redox balance in the brain and alters brain development and function. This study evaluated the effects of subcutaneous melatonin (MLT) administration, treadmill exercise (TE), and their combination on behavioral parameters (anxiety and memory), brain electrical activity (cortical spreading depression, CSD), and brain oxidative stress in well-nourished (*n* = 40) and malnourished (*n* = 40) young male rats.

**Methods:**

Both well-nourished and malnourished rats were assigned to treadmill exercise (*n* = 20) or sedentary (*n* = 20) groups. Each group (exercised and sedentary) received either MLT (subcutaneous; *n* = 10/group) or vehicle (*n* = 10/group). MLT (10 mg/kg on alternate days) and TE (forced running on a treadmill for 40-min daily sessions, 3 days/week) were conducted from P25 to P55. Immediately after TE and MLT treatments, animals underwent behavioral tests for anxiety (elevated plus maze and open field) and object recognition memory. CSD was continuously recorded for 4 h. Brains were collected for redox balance analyses.

**Results:**

Malnutrition increased anxiety-like behaviors, impaired memory, accelerated CSD propagation, and disrupted cortical redox balance. Both MLT administration and TE reduced these adverse effects, improving behavioral performance, slowing CSD, and attenuating prooxidant markers. The combined interventions produced values closer to those of well-nourished animals.

**Conclusion:**

The data suggest that melatonin and aerobic exercise have complementary neuroprotective effects in malnourished young rats, mitigating behavioral and electrophysiological disturbances and restoring brain oxidative balance. These interventions may be promising strategies for minimizing the consequences of early-life protein malnutrition on neurodevelopment.

## 1 Introduction

In the mammalian brain, malnutrition leads to several alterations, such as a decrease in the number of neurons in the parahippocampal region (Amaral et al., 2025), loss of striatal serotonin, degeneration of pyramidal cells in the hippocampus, and a marked reduction in cell density in the prefrontal cortex (Abey et al., 2024). Protein malnutrition can result in excessive production of free radicals and oxidative damage to macromolecules (Hegde et al., 2025). Thus, developing organisms exposed to malnutrition can experience significant functional and structural changes in the nervous system, including behavioral and electrophysiological alterations (Figueira-de-Oliveira et al., 2025).

Given the cellular and neurofunctional damage caused by redox imbalance induced by protein malnutrition, interventions that modulate the brain’s redox state are important. Alternatives that promote antioxidant actions, such as melatonin (MLT) administration (El Brouzi et al., 2024) and physical exercise (Sousa-Fernandes et al., 2024), are promising strategies to mitigate damage to the central nervous system. Both MLT and physical exercise can reduce reactive oxygen species levels and are therefore of particular interest in experimental models of neurometabolic vulnerability. The neurohormone MLT, synthesized by the pineal gland, is especially relevant in this context, as it provides cellular protection against oxidative stress (Reiter et al., 2016). MLT is an amphiphilic molecule that can cross the blood–brain barrier and act as a neuroprotective agent in conditions of neurodegeneration (Remigante et al., 2024). Thus, its action complements the protective effects of physical exercise, offering a promising approach to mitigate the neurological effects of protein malnutrition. However, there is still no evidence on how the combined effects of these interventions can influence brain electrophysiological functioning in organisms subjected to protein malnutrition.

Physical activity positively affects the nervous system, enhancing cognition, general arousal, and well-being (Gerber et al., 2025). Treadmill exercise stimulates the release of antioxidants in the brain, serving as a protective factor against the unregulated action of reactive oxygen species (Tung et al., 2024). In an adaptive context, the plasticity induced by physical exercise enables the central nervous system to acquire new functions in response to environmental changes (Mansoor et al., 2025), acting as a reprogramming agent that reduces the harmful effects of protein malnutrition.

In this context, the excitability-dependent brain phenomenon known as Cortical Spreading Depression (CSD) is a useful tool for analyzing electrophysiological parameters in the cerebral cortex (Guedes and Abadie-Guedes, 2019). This phenomenon consists of a wave of reduced spontaneous neuronal activity, triggered by an electrical, mechanical, or chemical stimulus at a single cortical point, involving intense ionic translocation between the intra- and extracellular compartments (Kraig and Nicholson, 1978), and is accompanied by vascular changes (Leăo, 1944a; Leăo, 1944b). The propagation of CSD can therefore be modulated by the environmental conditions to which the organism is subjected, as occurs in cases of malnutrition or physical exercise (Silva-Gondim et al., 2019).

Considering that both MLT and physical exercise have potentially neuroprotective properties, this study aimed to evaluate in rats how the combination of these two interventions modulates the neurophysiological effects of protein malnutrition, with emphasis on the propagation of CSD as a functional marker of cortical excitability. Additionally, the study investigated the potential effects of this combination (MLT and physical exercise) on anxiety-like behavior, memory, and brain redox balance. Our goal is to comprehensively understand the neuroprotective mechanisms of MLT and physical exercise against malnutrition during development.

## 2 Materials and methods

### 2.1 Animals

Our University’s Institutional Ethics Committee for the use of animals in scientific research approved this study (approval protocol no. 0096/2022), and its standards comply with those of the National Institutes of Health Guide for the Care and Use of Laboratory Animals (Bethesda, MD, USA). Eighty male Wistar rat pups (45 g ± 5 g; 25 days old) from the university vivarium were used. All animals were housed in an experimental room at 22 ± 2 °C and maintained on a reversed light–dark cycle (dark period from 8 a.m. to 8 p.m.). The animals had free access to water and food. All efforts were made to minimize animal suffering and to use the fewest possible animals to obtain valid results.

### 2.2 Experimental protocol

**Figure 1:**
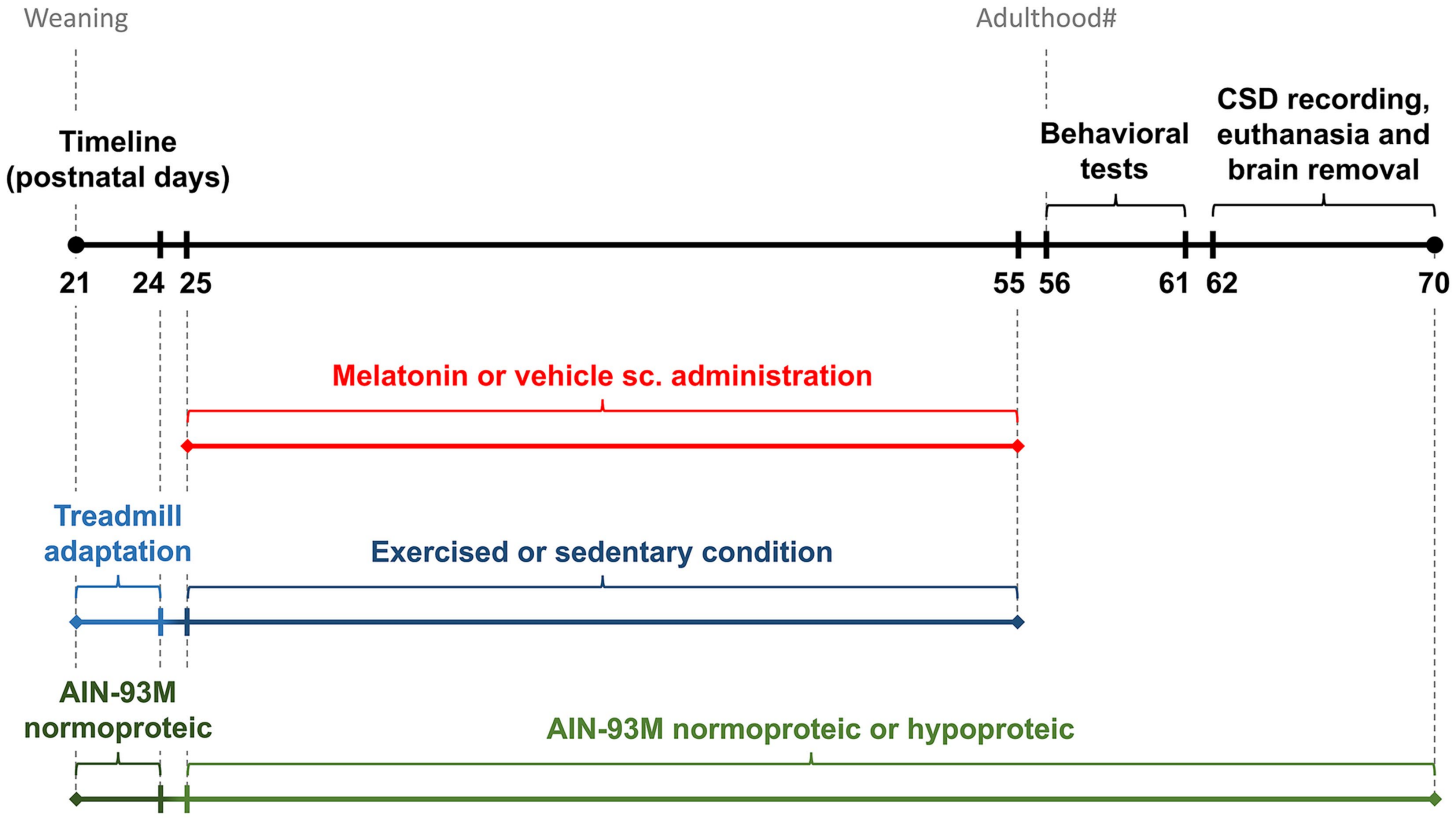
Timeline illustrating the experimental procedures for physical exercise, melatonin administration, nutritional manipulation, behavioral tests, and CSD recordings. ^#^The rat’s adulthood begins around the eighth week of life (Sengupta, 2013). SC, subcutaneous.

The rats were weaned on postnatal day 21 (P21) and housed in group cages. The animals were randomized into eight experimental groups, with 10 animals per group. From P21 to P24, the animals underwent dietary adaptation with a normal protein AIN-93 diet and physical exercise adaptation. From P25 to P55, the animals underwent MLT administration and physical exercise protocols. At the end of these protocols, the rats underwent behavioral testing from postnatal day 56 (P56) to P61, followed by electrophysiological recordings of CSD from P62 to P70. Nutritional manipulation with an AIN-93 M diet was maintained from P25 until the day of electrophysiological recording (Figure 1).

### 2.3 Experimental diets

The diets were formulated according to the guidelines of the American Institute of Nutrition (AIN) (Reeves et al., 1993). Wellnourished rats received the AIN-93 M diet containing 12% protein, while undernourished rats received a modified version of the same diet with 7% protein. Table 1 presents the composition of the experimental diets. Micronutrients (vitamins and minerals) were provided through a standardized mixture (AIN-93-MX and AIN-93-VX), previously obtained from Rhoster, Limited, in accordance with the nutritional guidelines established by the AIN protocol. For detailed information on these mixtures, refer to the Supplementary Tables 1, 2.

**Table 1:** Composition of the diets [Adapted from Reeves et al. (1993)].

Ingredients (g/100 g diet)	AIN-93 M	AIN-93 M
	Normoproteic	Hypoproteic
Cornstarch (87% carbohydrates)	46.57	52.26
Dextrinized Starch (92% tetrasaccharides)	15.50	15.50
Casein (≥ 85% proteins)	14.00	8.30
Sucrose	10.00	10.00
Soybean oil	4.00	7.00
Fibers	5.00	5.00
Mineral Mix (AIN-93G-MX)	3.50	3.50
Vitamin Mix (AIN-93-VX)	1.00	1.00
L-Methionine	0.18	0.18
Choline Bitartrate (41.1% choline)	0.25	0.25
Tert-butylhydroquinone	0.0008	0.0008
Caloric contribution of macronutrients (U/100 g diet)		
Total energy (kcal)	370.4	373.6
% as protein	12.95	8.88
% as carbohydrate	77.32	81.47
% as lipid	9.72	9.63

### 2.4 Melatonin (MLT) dosage and application

MLT (purchased from Sigma-Aldrich, St. Louis, United States) was administered from P25 to P55 on alternate days at a dose of 10 mg/kg body weight by subcutaneous injection at the beginning of the dark cycle (8 h), for a total of 15 days of application. This dose was determined *a priori* based on evidence from our laboratory, obtained in male Wistar rats during development, indicating that 10 mg/kg, compared to a higher dose, is associated with reduced CSD propagation and increased cortical markers of antioxidant capacity (Araújo et al., 2023). MLT was dissolved in 0.1 mL saline solution (0.9% NaCl) containing 5% ethanol (Araújo et al., 2023). The subcutaneous route was chosen because it is a safe method for administering drugs to animals, especially when gradual and sustained absorption is required. This route allows for relatively slow absorption, helping maintain constant plasma drug levels for a longer period. Additionally, it is less invasive than intramuscular, intraperitoneal, or intravenous routes, is easier to perform, causes less pain and stress to the animal, and carries a lower risk of complications compared to the other routes mentioned (Kagan et al., 2007; McDonald et al., 2010).

### 2.5 Exercise program

The animals were divided into two groups: sedentary and exercised. The experimental groups performed forced physical exercise on a motorized treadmill (INSIGHT, model EP-131) for rodents. This modality was chosen because it allows standardization of exercise intensity and frequency (Segabinazi et al., 2019). Initially, the animals underwent familiarization sessions, during which, for 3 days (P22-24), the rats were kept on the treadmill for 10 min with it turned off for visual and olfactory adaptation. Then, at a speed of 8 m/min, the rats remained active for 5 min, to adapt to sound and movement, according to González-Chávez et al. (2019). The physical exercise protocol was applied in moderate-intensity sessions from P25 to P55, lasting 40 min per session, following a protocol adapted from Braz et al. (2023). Each session included a 5-min warm-up at 12 ± 2 m/min, followed by 30 min at 20 ± 2 m/min, and a 5-min cool-down at 12 ± 2 m/min. The sessions always started at 10 a.m., with the treadmill kept at a flat incline (0°), three times a week. Forced treadmill sessions were conducted without electrical shocks; animals that refused to run were gently encouraged only with a wooden stick. If they continued to refuse, they were excluded from the study and accounted for in the final *n* = 10 per group. Animals in the sedentary group were placed on the treadmill for the same period, but the device remained off (Téglás et al., 2019). It is also important to state that forced treadmill exercise is considered a low-stress paradigm (Svensson et al., 2016). Since we used this method in previous studies and did not observe conspicuous signs of stress (e.g., changes in body weight) (Vitor-de-Lima et al., 2019), no physiological stress markers were collected in the present study. Further observations on this topic are discussed in the study limitations section.

### 2.6 Open field test (OF)

Each rat was placed in the center of the apparatus, a circular arena 90 cm in diameter surrounded by a circular wall 52 cm high. The OF device was in a room with dim red lighting and sound attenuation. Rat movements were recorded for 5 min using a digital camera. After each test, the OF apparatus was cleaned with 70% ethanol. Video-recorded activity was stored on a computer and later analyzed with ANYmaze™ software (version 4.99 m).

### 2.7 Object recognition tasks

Two object recognition tasks (ORTs) were conducted in the OF arena on two consecutive days, starting 24 h after the open-field test. Each ORT consisted of two sessions. In the first session (training phase), two identical objects were placed equidistant from the walls and symmetrically positioned around the arena’s center. Rats were allowed to explore the objects for 5 min. After a 40-min intersession interval, they returned for the second (test) session. During the test session on day 1, one of the objects was moved to a new spatial location (spatial recognition test). On day 2, one of the objects was replaced with a novel object of a different shape (novel object-shape recognition test). Based on the exploration times of the novel (N) and familiar (F) locations or objects, a discrimination index (DI) was calculated using the formula: DI = (TN – TF) / (TN + TF), where TN is the time spent exploring the novel object, and TF is the time spent exploring the familiar one.

### 2.8 Elevated plus maze (EPM) test

Twenty-four hours after the last object recognition task, the animals underwent the elevated plus maze (EPM) test. The EPM apparatus had a cross-shaped design, with four arms, each measuring 49 cm in length and 10 cm in width: two open arms and two closed arms (with side walls), arranged perpendicular to each other. All arms extended from a central square platform measuring 10 × 10 cm. At the start of the test, each animal was individually placed in the center of the maze, facing one of the open arms. Rats were allowed to explore the maze freely for 5 min under dim red lighting in a sound-attenuated room. Behavioral activity was recorded by a video camera, stored on a computer, and later analyzed using ANYmaze™ software (version 4.99 m). After each trial, the apparatus was cleaned with 70% ethanol.

### 2.9 Recording of cortical spreading depression

**Figure 2:**
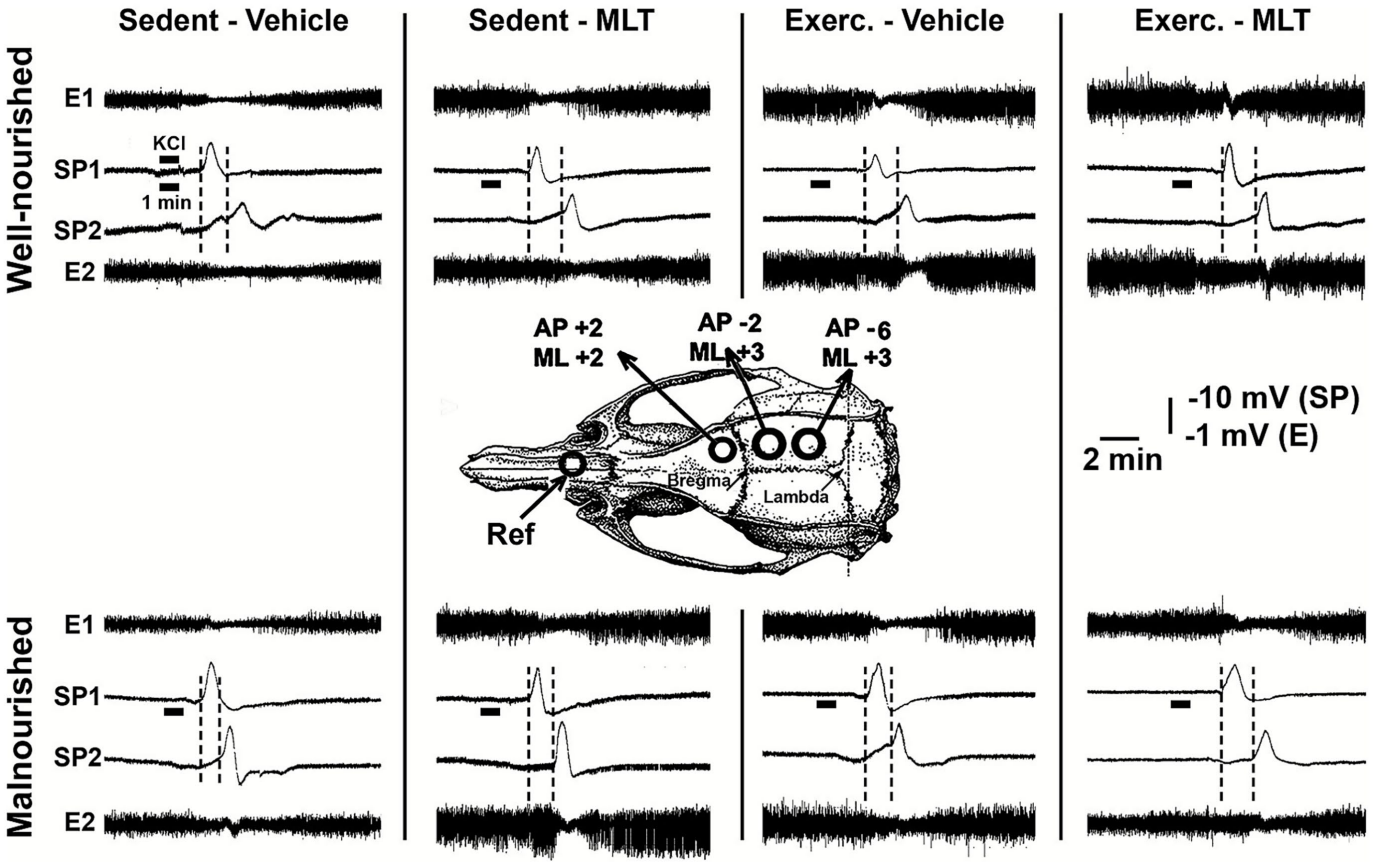
Electrophysiological recordings of CSD were obtained from four well-nourished and four malnourished rats, representing the eight treatment groups defined in the main text. The skull diagram shows the positions of the cortical recording electrodes, with the reference electrode (Ref) placed on the nasal bone. Bregma coordinates are given in millimeters along the anteroposterior (AP) and mediolateral (ML) axes. The diagram indicates the opening site used for KCl application to induce CSD in the frontal region (AP + 2/ML + 2) and the openings in the parietal region for placement of recording electrodes at positions 1 (AP –2/ML + 3) and 2 (AP –6/ML + 3). The slow potential change (SP) and the electrocorticogram (E) are also shown. The vertical dashed lines indicate the latency of a CSD wave as it crosses the interelectrode distance. This latency was shorter in the malnourished groups than in the well-nourished groups. Compared with control groups of the same nutritional status, the latencies in the exercised and/or MLT groups were longer. AP and ML coordinates are defined according to the rat stereotaxic atlas of Paxinos and Watson (2007).

Electrophysiological recording of CSD was performed 1 to 5 days after the behavioral tests. Animals were anesthetized intraperitoneally with a combination of urethane (1 g/kg) and alpha-chloralose (40 mg/kg), and three burr holes were made in the right side of the skull to expose portions of the cortical surface (bregma coordinates are given in millimeters along the anteroposterior (AP) and mediolateral (ML) axes, as shown in Figure 2). The first hole was drilled in the frontal bone (AP + 2/ML + 2), and the second and third holes in the parietal bone (AP –2/ML + 3 and AP –6/ML + 3, respectively). The frontal hole served as the site for applying the stimulus that triggered the CSD, which then propagated and was recorded through the parietal openings. CSD was induced every 20 min by placing a small cotton ball (1–2 mm in diameter) soaked in 2% potassium chloride (KCl) solution (approximately 270 mM) on the frontal opening for 1 min. For 4 hours, reductions in electrocorticogram (ECoG) amplitude and shifts in direct current (DC) potential associated with CSD were recorded using a digital acquisition system (MP-150, Biopac, USA) and the AcqKnowledge software (version 4.1) with 16-bit digitization and a sampling rate of 1.0 kHz per channel. To obtain DC, the signal was filtered at 0.0–300 Hz and amplified 50 times; while to obtain the ECoG, the signal was filtered at 0.5–35 Hz and amplified 5,000 times. This system allowed real-time visualization and storage on a computer. Throughout the recording session, rectal temperature was maintained at 37 ± 1 °C using an electric heating pad. The following CSD parameters were calculated: (1) mean propagation velocity, (2) amplitude, and (3) duration of the negative component of the slow potential shift.

### 2.10 Oxidative stress

Samples from the left (CSD-free) portion of the cerebral cortex were used to determine levels of lipid peroxidation and reduced glutathione (GSH). Lipid peroxidation was assessed by measuring thiobarbituric acid-reactive substances (TBARS) in tissue homogenates, following the method described by Ohkawa et al. (1979). Tissue samples were homogenized (5 g:1 mL) in phosphate-buffered saline (PBS) containing 137 mM NaCl, 2.7 mM KCl, 10 mM Na2HPO4, and 1.8 mM KH2PO4, supplemented with 0.15 mg/mL trypsin inhibitor (type II-S) and 1 mM PMSF, on ice. The homogenate was then centrifuged at 12,000 g for 12 min at 4 °C, and the supernatant (100 mg/mL) was added to a reaction medium containing 0.3% thiobarbituric acid, 0.4% SDS, and 7.5% acetic acid (pH 3.5). The mixture was heated to 95 °C for one hour. Samples were centrifuged (1,000 g for 10 min), and the absorbance of the supernatant was measured at 535 nm. TBARS concentration was calculated using 1,1,3,3-tetraethoxypropane as the standard. Data were corrected for the protein concentration of the homogenate.

The same supernatant obtained from the tissue homogenate was used to determine GSH levels by measuring non-protein sulfhydryl groups (Sedlak and Lindsay, 1968). The homogenate supernatant was treated with 1 volume of 10% trichloroacetic acid to precipitate proteins, then centrifuged at 5,000 g for 10 min. The resulting supernatant, containing non-protein sulfhydryl groups, was reacted with 4 mM 5,5′-dithiobis(2-nitrobenzoic acid) in a solution of 250 mM TRIS, 2.5 mM EDTA, and 10% methanol. GSH concentration was calculated using L-cysteine as the standard. Data were corrected for the protein concentration of the homogenate.

### 2.11 SOD and catalase activity

SOD activity was determined by assessing the tissue homogenate’s ability to decrease the formation of the pink chromophore (adrenochrome) produced by the oxidation of epinephrine (Misra and Fridovich, 1972). The sample (10 mg protein/mL) was added to a 50 mM sodium carbonate buffer, pH 10.2, and then supplemented with 3.0 mM epinephrine. The rate of adrenochrome formation was measured in triplicate by recording absorbance at 480 nm over 2 min, with readings every 15 s. SOD activity was expressed in arbitrary units, based on the capacity to reduce the spontaneous rate of adrenochrome formation, and was normalized to the protein content of the sample.

Catalase activity was determined by measuring the decrease in absorbance due to the reduction of H2O2 to water, as described by Aebi (1984). The sample (10 mg protein/mL) was added to 10 mM hydrogen peroxide in 50 mM potassium phosphate buffer (pH 7.0). After homogenization, the rate of H2O2 decomposition was measured at 240 nm every 15 s over 2 min. Samples were analyzed in triplicate, and values are expressed as mmol H2O2 consumed per minute, calculated using the H2O2 extinction coefficient and normalized to the sample’s protein content.

### 2.12 NADPH oxidase activity

NADPH oxidase activity was evaluated by quantifying superoxide produced in the presence of NADPH (100 μM), as described by Lima et al. (2021). Superoxide production was measured using lucigenin-derived chemiluminescence. Tissue homogenate samples (100 μg/mL) were added to a reaction medium containing 10 μM lucigenin and 100 μM NADPH diluted in PBS, and luminescence was measured with a luminometer. Results were expressed as relative light units (RLU) per minute and normalized to the protein content in the sample. Superoxide release was also measured in the absence of NADPH to represent basal superoxide anion production.

### 2.13 Sample size calculation

To ensure the samples were sufficient to detect the expected effect sizes, we performed sample size calculations using an alpha value of 5%, a power of 80%, and standard deviation estimates from previous studies in our laboratory (Guedes, 2011; Guedes and Abadie-Guedes, 2019; Figueira-de-Oliveira et al., 2025). All samples in this study met the calculated minimum requirement.

### 2.14 Statistical analysis

Results were expressed as mean ± standard deviation (SD). The statistical software used was Sigmastat® version 3.5. Intergroup differences were analyzed using a three-way ANOVA, with nutritional status (well-nourished versus malnourished), exercise condition (sedentary versus exercised), and systemic treatment (vehicle versus MLT) as factors. When appropriate, ANOVA was followed by the Holm-Sidak test. Differences with *p* < 0.05 were considered significant.

## 3 Results

### 3.1 Anxiety-like behavior

**Figure 3:**
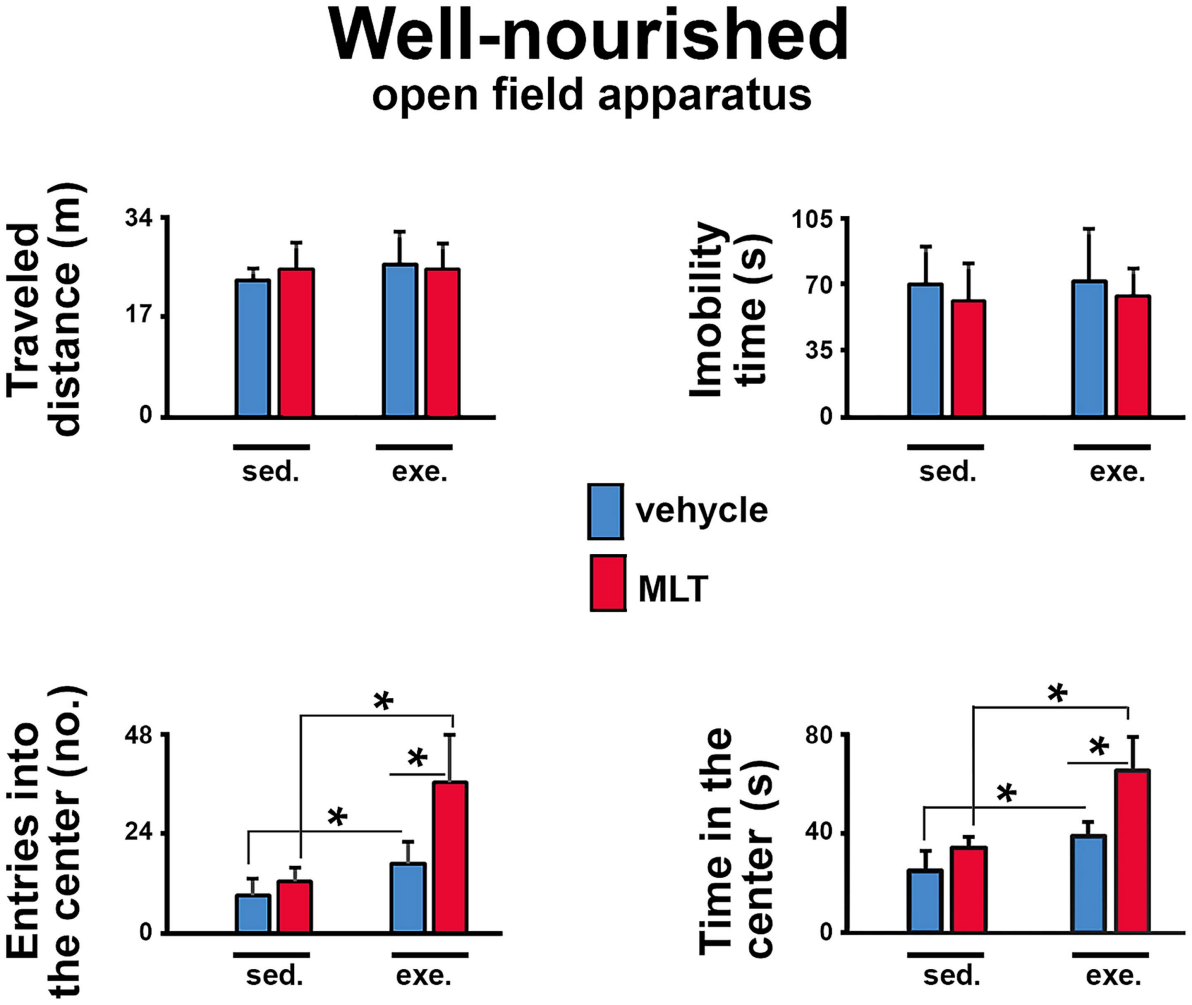
Behavioral responses in the open field (OF) test in well-nourished rats. The graphs show the traveled distance (m), the immobility time (s), the number of entries into the center (*n*), and the time in the center (s). Data are presented as mean ± standard deviation (SD) for 10 animals per group. *Indicates a significant difference between exercised and sedentary groups, as well as between animals treated with vehicle solution and melatonin (*p* < 0.001; three-way ANOVA followed by the Holm-Sidak *post hoc* test).

**Figure 4:**
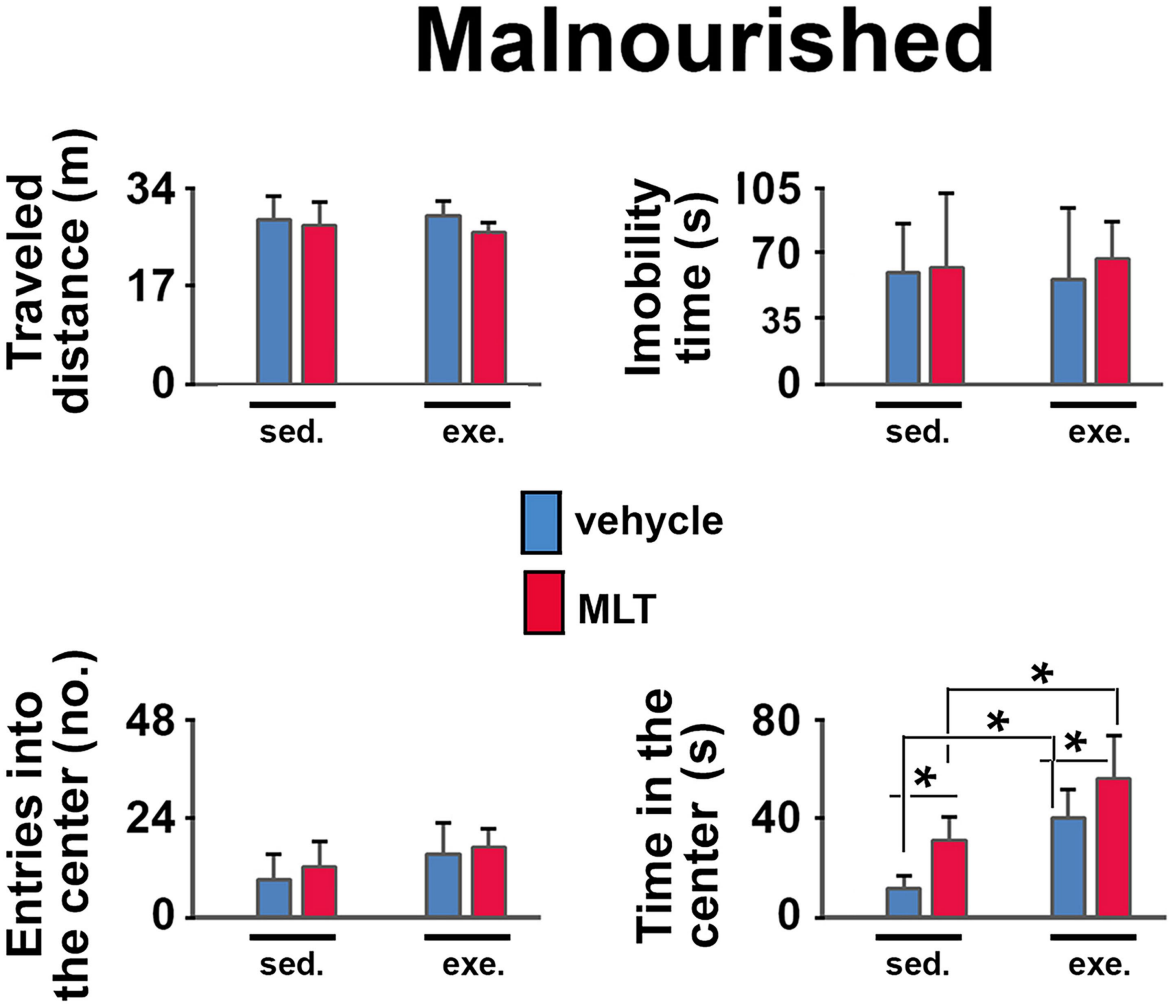
Behavioral responses in the open field (OF) test in malnourished rats. The graphs show the traveled distance (m), the immobility time (s), the number of entries into the center (*n*), and the time in the center (s). Data are presented as mean ± SD for 10 animals per group. *Indicates a significant difference between exercised and sedentary groups, as well as between animals treated with vehicle solution and melatonin (*p* < 0.001; three-way ANOVA followed by the Holm-Sidak *post hoc* test).

In the open field test (OF), well-nourished animals (Figure 3) showed that physical exercise led to more entries and longer stays in the central area of the apparatus compared to sedentary animals. This pattern was also seen in exercised rats treated with melatonin compared to those given only a vehicle solution. In the malnourished groups (Figure 4), exercised animals also had more entries and longer stays in the central region, indicating an anxiolytic effect of exercise. Furthermore, MLT administration further enhanced this effect, as MLT-treated rats performed better than vehicle-treated rats.

**Figure 5:**
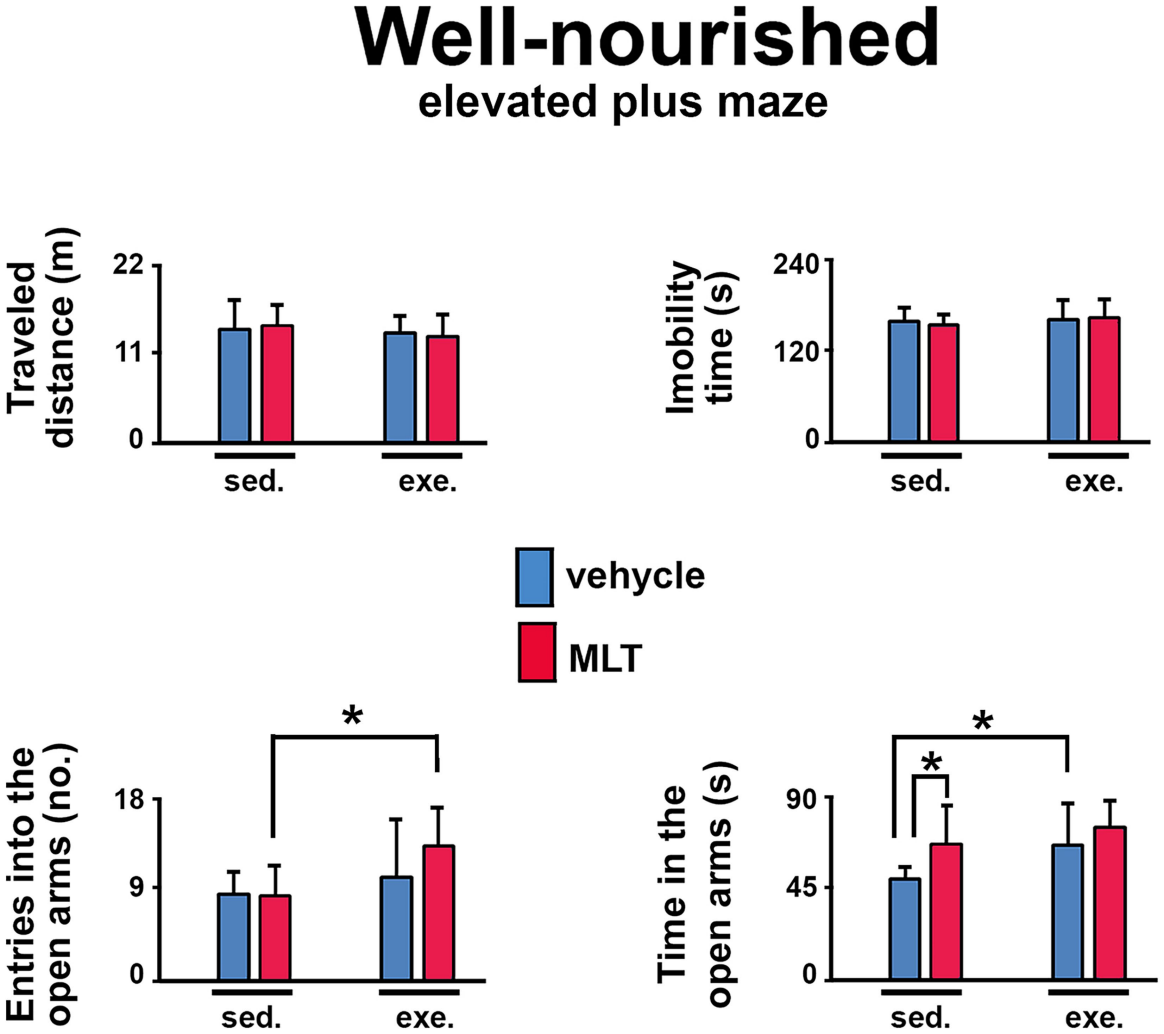
Behavioral responses in the elevated plus maze (EPM) test in well-nourished rats. The graphs show the traveled distance (m), immobility time (s), entries into the open arms (*n*), and time in the open arms (s). Data are presented as mean ± SD for 10 animals per group. *Indicates a significant difference between exercised and sedentary groups, as well as between animals treated with vehicle solution and melatonin (*p* < 0.001; three-way ANOVA followed by the Holm-Sidak *post hoc* test).

**Figure 6:**
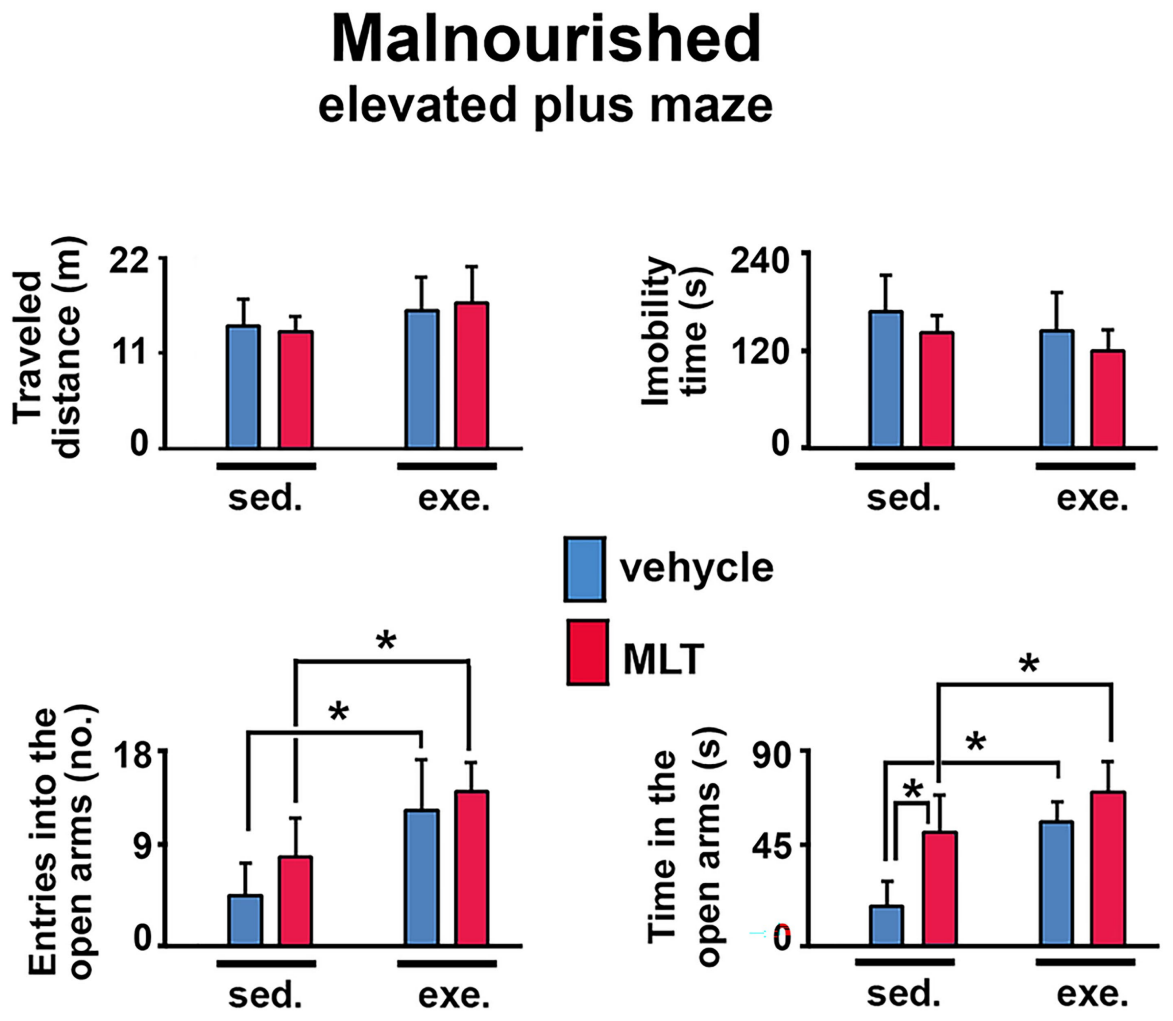
Behavioral responses in the elevated plus maze (EPM) test in malnourished rats. The graphs show the traveled distance (m), immobility time (s), entries into the open arms (*n*), and time in the open arms (s). Data are presented as mean ± SD for 10 animals per group. *Indicates a significant difference between exercised and sedentary groups, as well as between animals treated with vehicle solution and melatonin (*p* < 0.001; three-way ANOVA followed by the Holm-Sidak *post hoc* test).

The elevated plus maze (EPM) task was also used to assess anxiety behavior in well-nourished (Figure 5) and malnourished (Figure 6) animals. In both groups, exercised animals made more entries into and spent more time in the maze’s open arms than sedentary animals. This effect was more pronounced in the malnourished groups, suggesting that these animals were more sensitive to the anxiolytic effects of exercise.

### 3.2 Object recognition memory

**Figure 7:**
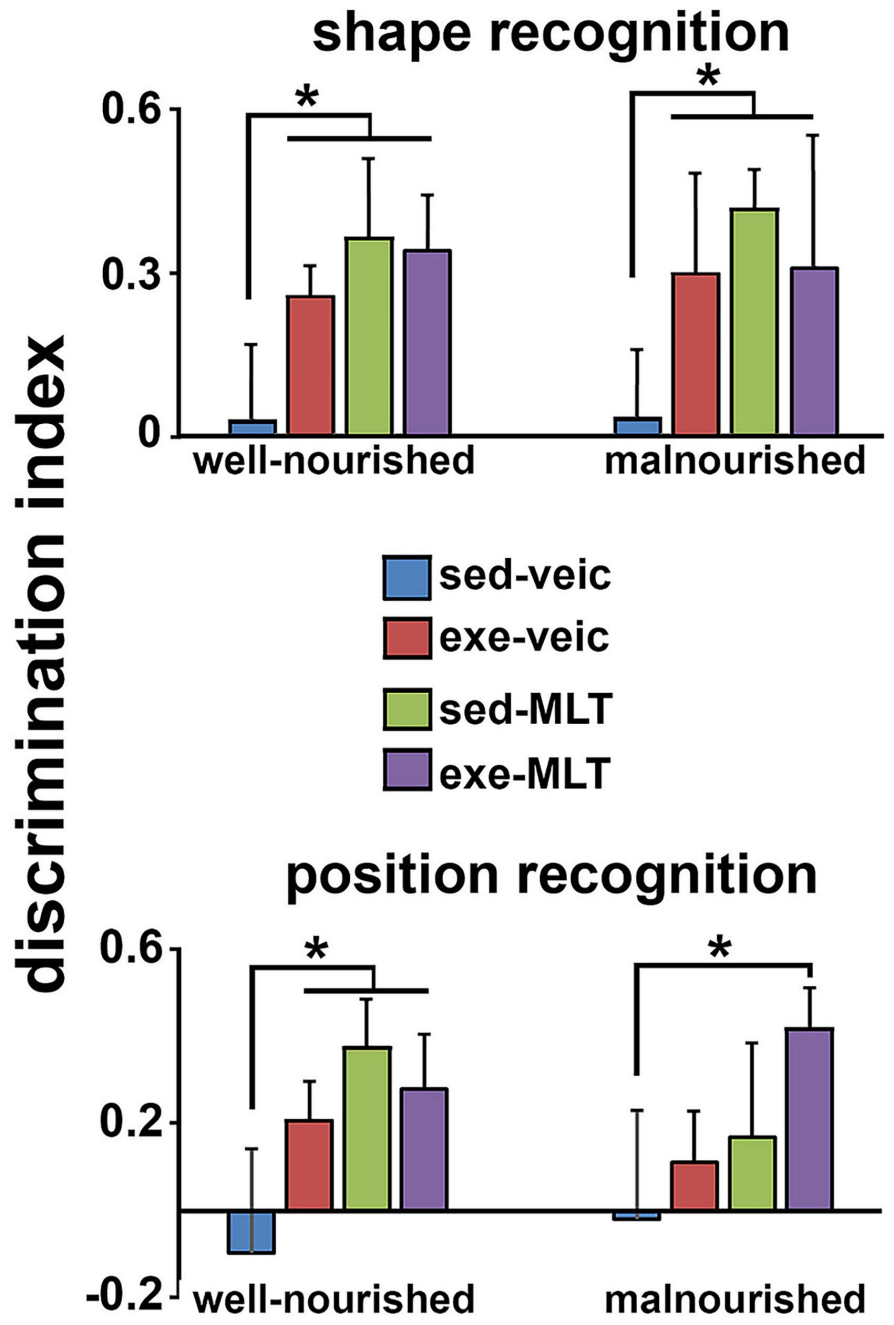
Discrimination index (adimensional parameter) in shape and position recognition tests in well-nourished and malnourished animals. Data are presented as mean ± standard deviation. *Indicates a significant difference compared to the control group (sedentary + vehicle) within the same nutritional condition (*p* < 0.001; three-way ANOVA followed by the Holm-Sidak *post hoc* test).

Short-term memory was assessed using the object recognition test in two modalities: shape recognition and position recognition. Performance was measured by the discrimination index, defined as the proportion of time spent exploring the novel object relative to the total time spent exploring all objects. Positive values of this index indicate a preference for the novel object, reflecting better recognition memory performance. The results showed that animals subjected to physical exercise and/or treated with melatonin had higher discrimination indices than the sedentary group treated with a vehicle. In both the shape and position recognition tests, the exercised and/or melatonin-treated groups performed better than the sedentary control group, suggesting that both treatments facilitated recognition of the novel stimulus (Figure 7).

### 3.3 Parameters of cortical spreading depression

**Figure 8:**
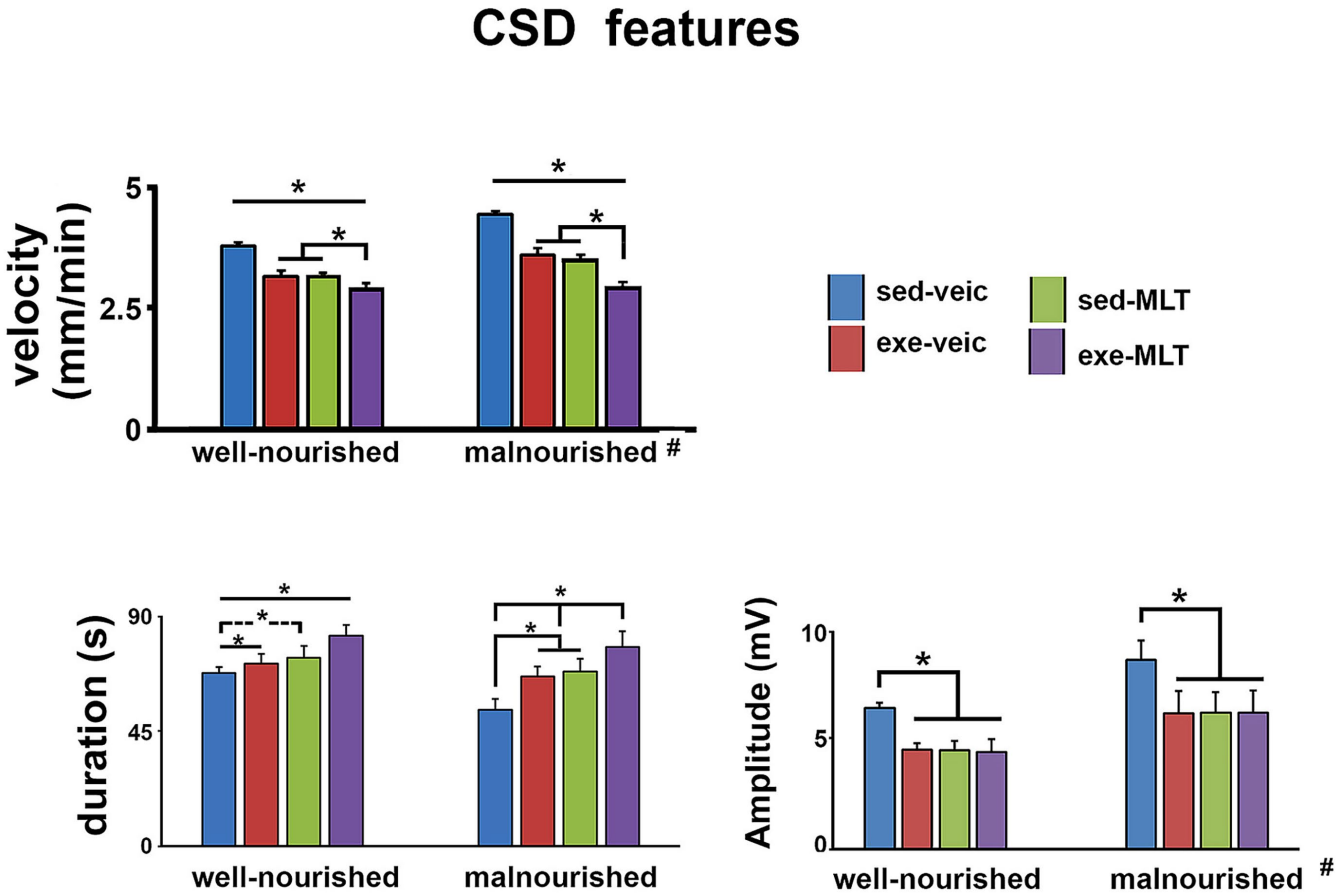
CSD features in well-nourished and malnourished rats. The graphs show the propagation speed (mm/min), duration (s), and amplitude (mV) of the CSD event, recorded in the eight experimental groups. Data are presented as mean ± standard deviation, *n* = 10 animals per group. *Indicates a significant difference between groups within the same nutritional condition; #Indicates significant difference between well-nourished and malnourished vehicle groups (*p* < 0.001; three-way ANOVA followed by the Holm-Sidak test); see Table 2.

After topical application of 2% KCl to the cortical region, CSD was confirmed by slow depolarization and reduced ECoG activity (Figure 2). Malnutrition and sedentary lifestyles enhanced CSD propagation compared with well-nourished, physically active groups. In contrast, both physical exercise and melatonin treatment showed neuroprotective effects, acting as antagonists of the phenomenon’s propagation. Animals subjected to physical activity and/or treated with melatonin exhibited a significant reduction in the speed and amplitude of CSD propagation, along with an increase in its duration (see Figure 8).

### 3.4 Redox balance in the cerebral cortex

**Figure 9:**
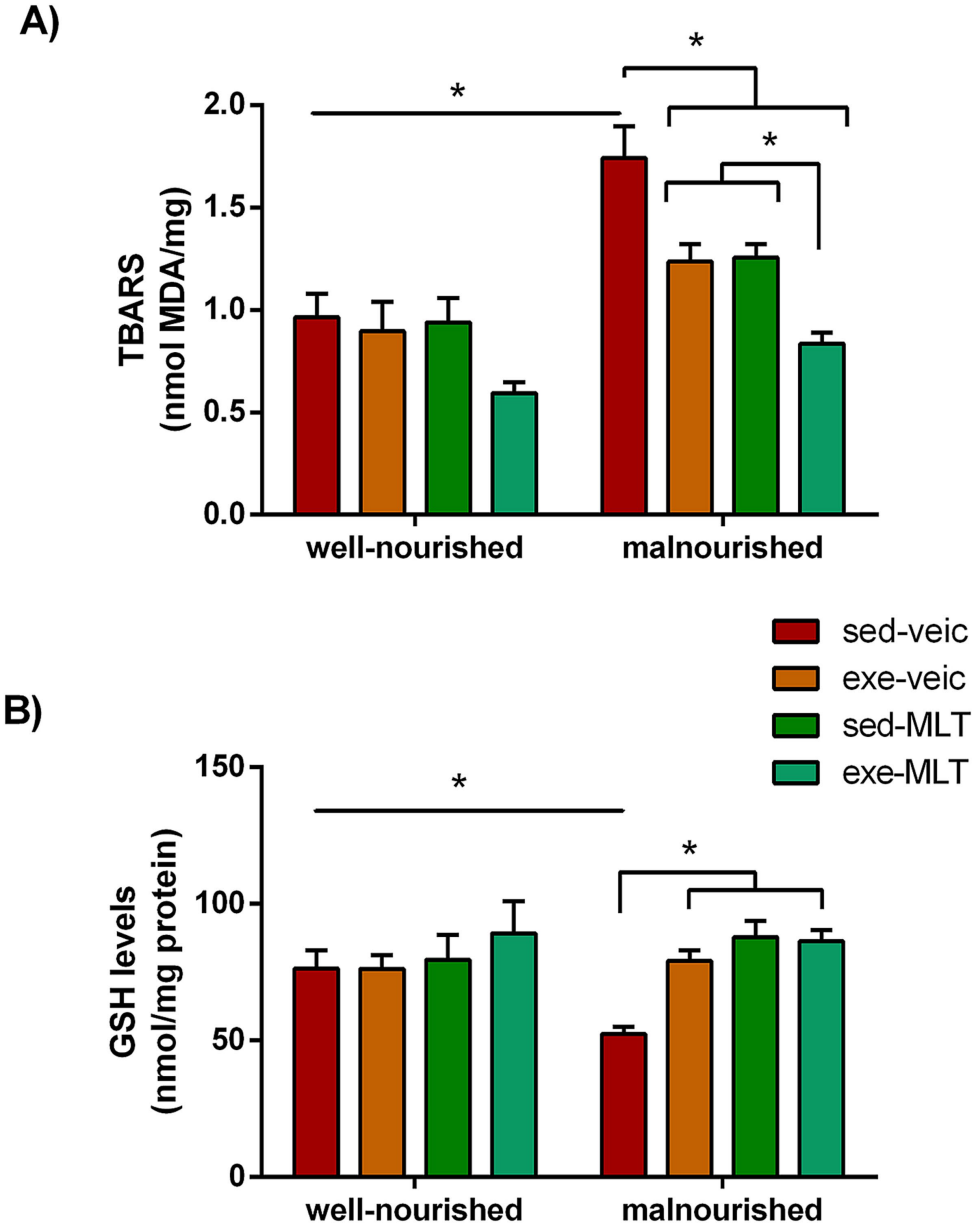
Lipid peroxidation and reduced glutathione (GSH) levels were evaluated in the cerebral cortex of well-nourished and malnourished animals that were subjected to vehicle (red, sedentary; orange, exercised), or to melatonin (MLT; dark-green, sedentary; light-green, exercised). **(A)** Lipid peroxidation was estimated by measuring thiobarbituric acid reactive substances (TBARS, in nmol MDA/mg protein). **(B)** GSH levels (in nmol/mg protein) were determined by measuring non-protein sulfhydryl groups. Data are presented as mean ± standard deviation. The sample size (*n*) ranged from 5 to 10. *Indicates a significant difference between experimental conditions (*p* < 0.001; three-way ANOVA followed by the Holm-Sidak *post hoc* test).

A low-protein diet disrupted cerebral redox balance. The malnourished-sedentary group treated with vehicle showed higher TBARS levels than the corresponding well-nourished group (well-nourished sedentary vehicle), indicating greater lipid peroxidation and oxidative damage (Figure 9A). In contrast, all malnourished groups subjected to physical exercise, melatonin (MLT) treatment, or both, had lower cerebral cortex TBARS levels than the malnourished sedentary vehicle group. The low-protein diet also significantly reduced glutathione (GSH) levels in the cerebral cortex of sedentary, malnourished rats compared to well-nourished controls (Figure 9B). Melatonin increased these GSH levels, and exercise produced a similar improvement. The combination of melatonin and exercise brought the values even closer to those observed in well-nourished animals, indicating a more robust restoration of the antioxidant system.

**Figure 10:**
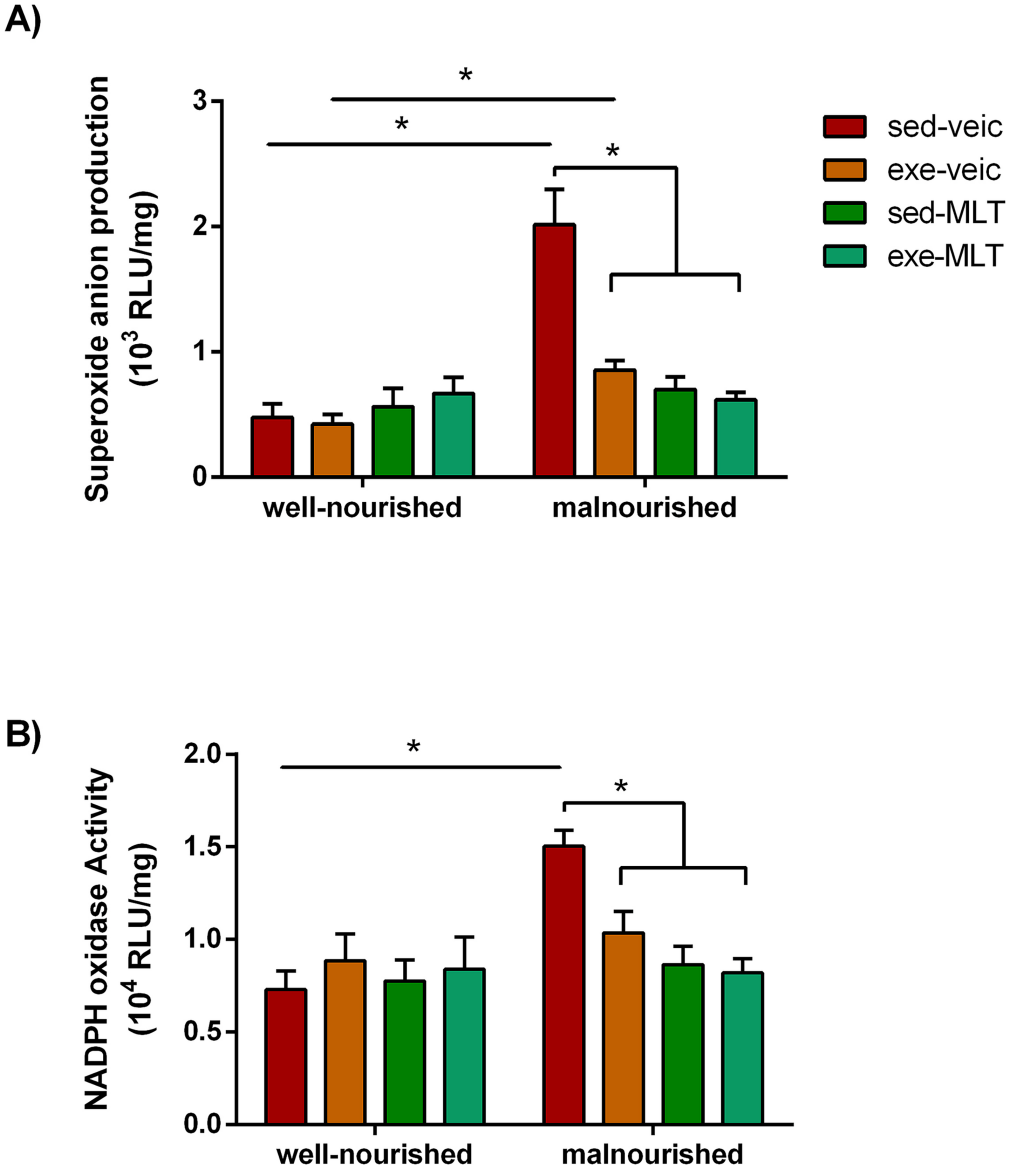
Evaluation of superoxide anion production **(A)** and NADPH oxidase activity **(B)** in well-nourished and malnourished rats. NADPH oxidase activity (in 10^4^ RLU/mg protein) was assessed by quantifying superoxide produced in the presence of NADPH (100 μM). Superoxide production (in 10^3^ RLU/mg protein) was measured using lucigenin-derived chemiluminescence. Data are presented as mean ± standard deviation. The sample size (*n*) ranged from 5 to 10. *Indicates a significant difference between experimental conditions (*p* < 0.001; three-way ANOVA followed by the Holm-Sidak *post hoc* test).

Basal production of superoxide anions in the cerebral cortex was significantly increased in the malnourished sedentary vehicle group compared to the well-nourished groups, confirming a basal pro-oxidant state (Figure 10A). In the tissue, NADPH oxidase activity, a major source of reactive oxygen species (ROS), was also elevated due to malnutrition. Both melatonin administration and physical exercise alone effectively reduced basal superoxide anion production and mitigated the hyperactivity of NADPH oxidase, bringing the levels closer to those of the well-nourished groups (Figure 10B). The combination of exercise and melatonin did not further inhibit basal superoxide anion production or NADPH oxidase activity in malnourished rats (Figure 10).

**Figure 11:**
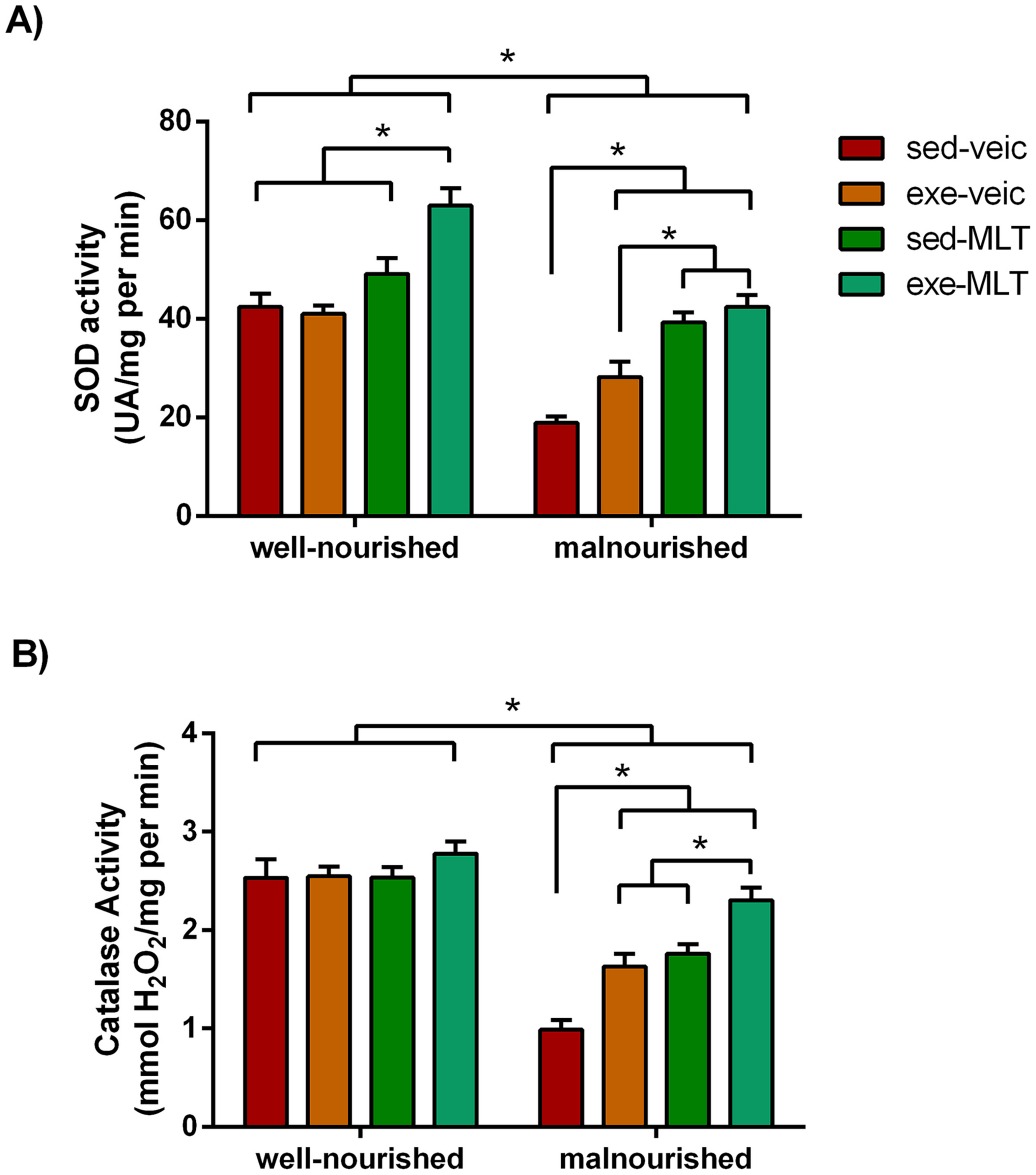
Activity of the antioxidant enzymes superoxide dismutase **(A)** and catalase **(B)** in well-nourished and malnourished rats. SOD activity (in AU/mg protein/min) was estimated by the ability of the tissue homogenate to decrease the formation of the pink chromophore (adrenochrome) resulting from the oxidation of epinephrine. Catalase activity (in mmol H_2_O_2_/mg protein/min) was estimated by measuring the decrease in absorbance caused by the reduction of H_2_O_2_ to water. Data are presented as mean ± standard deviation. The sample size (*n*) ranged from 5 to 10. *Indicates a significant difference between experimental conditions (*p* < 0.001; three-way ANOVA followed by the Holm-Sidak *post hoc* test).

Superoxide dismutase (SOD) activity was reduced in the cerebral cortex of the malnourished sedentary vehicle group compared to the well-nourished group (Figure 11A). In malnourished rats, physical exercise increased SOD activity than in the corresponding sedentary group. Additionally, both sedentary and exercised malnourished groups treated with melatonin showed greater SOD activity than their respective vehicle-treated groups. In the well-nourished groups, melatonin administration in the exercised group also increased SOD activity compared to the other well-nourished groups (Figure 11A).

Catalase activity was also reduced in the cerebral cortex of the malnourished sedentary vehicle group relative to the wellnourished sedentary vehicle group (Figure 11B). In malnourished rats, exercise and/or melatonin treatment led to greater catalase activity than in the malnourished sedentary vehicle group. However, the malnourished group that received both exercise and melatonin exhibited the highest catalase activity compared to the malnourished groups subjected exclusively to exercise or MLT treatment (Figure 11B).

A summary of all statistical differences is shown in Table 2.

**Table 2:** *F* and *p* values indicating the effects of the exercise (EX), malnutrition (MN), and melatonin administration (MLT) in young rats (three-way ANOVA followed by the Holm-Sidak test).

	Malnutrition	Exercise (2)	Melatonin (3)	Interaction 1 vs. 2	Interaction 1 vs. 3	Interaction 2 vs. 3	Interaction 1 vs. 2 vs. 3
OF-Traveled distance	*F*(7, 59) = 12.095; *p* = 0.001	*F*(7, 59) = 0.714; *p* = 0.401	*F*(7, 59) = 1.010; *p* = 0.319	No	No	No	No
OF-Immobility time	*F*(7, 59) = 0.671; *p* = *0*.416	*F*(7, 59) = 0.034; *p* = 0.853	*F*(7, 59) = 0.021; *p* = 0.885	No	No	No	No
OF-Entries into the center	*F*(7, 59) = 10.172; *p* = 0.002	*F*(7, 59) = 41.855; *p* < 0.001	*F*(7, 59) = 18.004; *p* < 0.001	Yes	Yes	Yes	Yes
OF-Time in the center	*F*(7, 59) = 5.697; *p* = 0.02	*F*(7, 59) = 96.981; *p* < 0.001	*F*(7, 59) = 49.425; *p* < 0.001	No	No	No	Yes
EPM-Traveled distance	*F*(7, 55) = 2.613; *p* = 0.112	*F*(7, 55) = 1.133; *p* = 0.292	*F*(7, 55) = 0.012; *p* = 0.913	Yes	No	No	No
EPM- Immobility time	*F*(7, 55) = 5.012; *p* < 0.029	*F*(7, 55) = 1.393; *p* = 0.243	*F*(7, 55) = 3.232; *p* = 0.078	No	No	No	No
EPM- Entries into the open arms	*F*(7, 55) = 0.018; *p* = 0.895	*F*(7, 55) = 34.630; *p* < 0.001	*F*(7, 55) = 3.836; *p* = 0.055	No	No	No	No
EPM-Time in the open arms	*F*(7, 55) = 16.543; *p* < 0.001	*F*(7, 55) = 30.078; *p* < 0.001	*F*(7, 55) = 23.872; *p* < 0.001	Yes	No	No	No
Object recogn. task (shape)	*F*(7, 59) = 0.253; *p* = 0.617	*F*(7, 55) = 33.638; *p* < 0.001	*F*(7, 55) = 6.527; *p* = 0.014	No	No	Yes	No
Object recogn. task (position)	*F*(7, 59) = 0.552; *p* = 0.461	*F*(7, 59) = 48.583; *p* < 0.001	*F*(7, 59) = 13.357; *p* < 0.001	No	No	Yes	Yes
CSD velocity	*F*(7, 72) = 346.406; *p* < 0.001	*F*(7, 72) = 893.790; *p* < 0.001	*F*(7, 72) = 842.756; *p* < 0.001	Yes	Yes	Yes	No
CSD amplitude	*F*(7, 72) = 120.552; *p* < 0.001	*F*(7, 72) = 44.635; *p* < 0.001	*F*(7, 72) = 36.029; *p* < 0.001	No	No	Yes	No
CSD duration	*F*(7, 72) = 51.032; *p* < 0.001	*F*(7, 72) = 142.363; *p* < 0.001	*F*(7, 72) = 87.328; *p* < 0.001	Yes	Yes	No	No
TBARs levels	*F*(7, 50) = 28.387; *p* < 0.001	*F*(7, 50) = 18.128; *p* < 0.001	*F*(7, 50) = 14.951; *p* < 0.001	No	No	No	No
GSH levels	*F*(7, 51) = 0.859; *p* = 0.358	*F*(7, 51) = 4.148; *p* = 0.047	*F*(7, 51) = 11.875; *p* = 0.001	No	No	No	Yes
Superoxide anion production	*F*(7, 59) = 29.237; *p* < 0.001	*F*(7, 59) = 9.879; *p = 0.003*	*F*(7, 59) = 10.410; *p* = 0.002	Yes	Yes	Yes	Yes
NADPH oxidase activity	*F*(7, 54) = 7.667; *p* = 0.008	*F*(7, 54) = 0.481; *p* = 0.491	*F*(7, 54) = 7.220; *p* = 0.010	No	Yes	No	No
SOD activity	*F*(7, 56) = 80.543; *p* < 0.001	*F*(7, 56) = 11.231; *p = 0.001*	*F*(7, 56) = 73.407; *p* < 0.001	No	No	No	Yes
Catalase activity	*F*(7, 57) = 99.218; *p* < 0.001	*F*(7, 57) = 15.239; *p* < 0.001	*F*(7, 57) = 20.436; *p* < 0.001	Yes	Yes	No	No

OF, open field test; EPM, elevated plus-maze test; CSD, cortical spreading depression; TBARs, thiobarbituric acid reactive species; GSH, glutathione; NADPH oxidase, nicotinamide adenine dinucleotide phosphate oxidase; SOD, superoxide dismutase.

## 4 Discussion

During the critical period of brain development – known as the “brain growth spurt” – fundamental processes for the maturation of the central nervous system occur, including neurogenesis, gliogenesis, synaptogenesis, and myelination (Corrigan et al., 2025). At this stage, the brain is highly susceptible to external insults (Guedes et al., 2012), with oxidative stress as a primary risk factor (Ribeiro-Carvalho and Krahe, 2023). Oxidative stress, resulting from an imbalance between ROS production and the body’s antioxidant capacity, can severely compromise the organization and function of the developing brain through biochemical and morphological changes (Lubrano et al., 2024).

Malnutrition, as a metabolic stress factor, is directly associated with the onset of neurodegenerative processes (Sato et al., 2021). Protein restriction increases the expression of inflammatory markers and induces structural and functional changes in neurons, impairing myelination, synaptic density, and neurotransmitter activity (Benevides et al., 2022; Samia et al., 2025). Abrupt reductions in protein intake further intensify oxidative stress, amplifying neuronal damage and enhancing the progression of neurological dysfunctions (Belchior et al., 2021).

Beyond its structural impact, malnutrition also affects behavior and brain excitability. Nutritional changes have been linked to anxious behavior, cognitive deficits, and altered brain excitability (Figueira-de-Oliveira et al., 2025). Artiukhov et al. (2022) demonstrated that redox imbalance is directly related to the manifestation of anxious behavior, reinforcing the role of oxidative stress in neurological and behavioral dysfunction.

In this context, the results of the present study show that both physical exercise and melatonin administration can mitigate the adverse effects of protein malnutrition, resulting in significant improvements in anxiety-like behavior, short-term memory retention, and electrophysiological activity. Animals subjected to these interventions exhibited reduced propagation of CSD, better performance in memory tests (object recognition), and anxiolytic-like behavioral responses (open field test and elevated plus maze).

CSD results from an intense wave of neuronal and glial depolarization that propagates slowly and is self-reproducing, accompanied by a temporary inhibition of brain electrical activity (Leăo, 1944a; Guedes, 2011). This phenomenon is directly involved in the pathogenesis of migraine aura and is considered the neurophysiological correlate of the sensory symptoms that precede the painful phase of this neurological condition (McLeod et al., 2025). Due to its clinical relevance, CSD has been widely used as an experimental model for screening therapeutic agents, allowing evaluation of the efficacy of drugs and nonpharmacological interventions in modulating cortical excitability and preventing the spread of this phenomenon (Chen and Ayata, 2017). Our findings align with previous evidence demonstrating the beneficial effects of physical exercise on both mental and physiological health in humans and animal models (Tari et al., 2025; He et al., 2025). Physical activity has been shown to improve anxiety-related behaviors, enhance memory, and slow the propagation of CSD, regardless of exercise type or life stage (Braz et al., 2023; Silva-Gondim et al., 2019; Vitor-De-Lima et al., 2024).

Mechanisms underlying these benefits include the induction of neurogenesis, angiogenesis, and synaptogenesis in the hippocampus – processes essential for improving memory and spatial learning (Kraemer and Kraemer, 2023; Zang et al., 2023). These processes are mediated by neurotrophins and growth factors, such as BDNF, VEGF, and IGF-1 (Khalil, 2024), which enhance cerebral blood flow and neuronal communication efficiency, thereby reducing brain excitability and slowing CSD propagation (Yen et al., 2023; Lima-De-Castro et al., 2025).

Melatonin has also proven to be an effective intervention. Studies from our group have demonstrated that neonatal administration of melatonin (10 mg/kg) slows CSD and improves behavioral parameters related to anxiety and memory (Araújo et al., 2023). The present investigation builds on these findings by showing that melatonin maintained its beneficial effects on electrophysiological and behavioral activity even in young rats subjected to malnutrition – a topic still unexplored in the literature. Melatonin’s neuroprotective action is largely attributed to its antioxidant, anti-inflammatory, and mitochondrial-preserving properties (Sieminski et al., 2024). At the cellular level, melatonin neutralizes free radicals, protects mitochondria from oxidative damage, and regulates immune responses (Huang et al., 2022). A recent study by Yu et al. (2024) showed that melatonin exerts neuroprotective effects against ischemic stroke-related damage by reducing iron overload and inhibiting ferroptosis—an iron-dependent form of cell death (Tuo et al., 2022).

Furthermore, melatonin has demonstrated efficacy in models of inflammatory, autoimmune, and neurodegenerative diseases. Shen et al. (2024) observed a significant anti-inflammatory effect in a murine model of psoriasis. Panmanee et al. (2025) reported mitochondrial protection in SH-SY5Y cells exposed to the Aβ₄₂ peptide, simulating Alzheimer’s-like pathology. Escribano et al. (2022) documented clinical improvement and a reduction in oxidative stress and intestinal dysbiosis in the experimental autoimmune encephalomyelitis (EAE) model—a well-established preclinical model for studying multiple sclerosis and other demyelinating diseases of the central nervous system.

Such effects have also been observed in humans. Gandolfi et al. (2020) reported improved sleep quality in ICU patients following melatonin supplementation. Palmer et al. (2020) found that women undergoing chemotherapy for breast cancer experienced reduced cognitive and depressive side effects when treated with melatonin. These findings indicate that, beyond its chronobiological role, melatonin can act as a functional modulator of neural activity, promoting neurochemical balance and synaptic plasticity in brain regions involved in cognition. These mechanisms support melatonin’s significant neuroprotective potential, particularly in preventing neuronal apoptosis, reducing ischemia/reperfusion damage, and attenuating neurodegenerative processes (Hu et al., 2023; Zhi et al., 2020). Based on this evidence, it is plausible to suggest that the effects of physical exercise and melatonin on the behavioral and electrophysiological parameters analyzed in this study are, at least in part, due to the positive modulation of endogenous antioxidant capacity. We propose that these interventions can reduce oxidative stress and its harmful effects on behavior and cortical electrical activity, even under adverse conditions such as early-life protein malnutrition.

### 4.1 Study limitations

Pharmacological evidence from our laboratory supports the plausibility that the cortical oxidative environment can modulate CSD dynamics in a dose-dependent manner. Araújo et al. (2023) observed divergent effects of melatonin depending on the dose: 10 mg/kg was associated with a profile more compatible with an antioxidant effect, slowing CSD and improving behavioral performance, whereas a higher dose showed the opposite effect on CSD propagation. Similarly, Mendes-da-Silva et al. (2014) demonstrated that ascorbic acid can exert antioxidant or pro-oxidant actions depending on the dose, with divergent effects on oxidative stress and CSD. In the present study, the melatonin dose selected for its antioxidant profile was associated with behavioral improvement and increased cortical resistance to CSD, reinforcing the notion that strengthening endogenous antioxidant capacity helps reduce deleterious effects associated with a pro- oxidant context, such as malnutrition. However, because this is a comparative design, our data do not establish causality. Future studies should employ specific mechanistic manipulations (e.g., pro-oxidant challenges in well-nourished animals, blocking antioxidant pathways in the exercise group, and independent modulation of cortical excitability) to test whether changes in redox state and/or CSD are necessary and sufficient for behavioral changes.

Although we used shock-free treadmill running with an adaptation phase, and previous studies from our laboratory have already observed a reducing effect of physical exercise on CSD propagation and anxiety-like behavior regardless of the paradigm adopted (forced or voluntary) (Braz et al., 2023; Silva-Gondim et al., 2019; Vitor-De-Lima et al., 2024), we recognize that imposed exercise can, under certain conditions, act as a stressor and influence behavioral outcomes (Svensson et al., 2016). However, in the present study, we did not observe anxiogenic effects of this intervention compared to sedentary groups. Future studies should include direct measures of stress (e.g., corticosterone or other physiological markers), as well as comparisons between imposed and voluntary exercise, to more accurately dissociate the effects of exercise from the stress component associated with the method.

Finally, in this study, only male rats were used as a common strategy to avoid modulation of the CSD phenomenon by natural fluctuations in estrogens and progestogens (Accioly and Guedes, 2020; Kudo et al., 2023). However, we recognize sex-related differences in redox balance (Kamper et al., 2009) and melatonin pharmacodynamics (Adamah-Biassi et al., 2014). Therefore, we acknowledge this limitation and recommend that future studies include both sexes.

## 5 Conclusion

The data presented in this study show that both physical exercise and melatonin administration exert significant neuroprotective effects against behavioral and electrophysiological deficits caused by early-life protein malnutrition. The interventions reduced oxidative stress, decreased the propagation of cortical spreading depression, and improved memory and anxiety-related behavioral parameters. These findings expand current knowledge of non-pharmacological strategies that can modulate cortical excitability and restore impaired neural functions in contexts of nutritional vulnerability during neurodevelopment. Continuing this line of research may significantly contribute to the development of integrative therapeutic approaches in nutritional neuroscience.

## Data Availability

The raw data supporting the conclusions of this article will be made available by the authors, upon request under reasonable grounds, for scientific research purposes only.

## Glossary

AIN: American Institute of Nutrition
AIN-93M: Maintenance diet formulated by AIN
ANOVA: Analysis of Variance
AP: Anteroposterior
CSD: Cortical spreading depression
DC: Direct current
DI: Discrimination index
ECoG: Electrocorticogram
EPM: Elevated plus maze
EX: Exercise
GSH: Glutathione
MDA: Malondialdehyde
ML: Mediolateral
MLT: Melatonin
MN: Malnutrition
NADPH: Nicotinamide adenine dinucleotide phosphate
OF: Open field
ORT: Object recognition task
P: Postnatal day
PBS: Phosphate-buffered saline
RLU: Relative light units
ROS: Reactive oxygen species
SC: Subcutaneous
SD: Standard deviation
SOD: Superoxide dismutase
TBARS: Thiobarbituric acid reactive substances
TE: Treadmill exercise
