# Supplementary material for: Physical exercise/melatonin interaction in young rats fed a low-protein diet: a behavioral, electrophysiological, and redox balance analysis
Source: Front Aging Neurosci. 2026 Feb 6;18:1740062. doi: 10.3389/fnagi.2026.1740062 (PMC12920428; doi:10.3389/fnagi.2026.1740062)
Supplement: Supplementary file 1 [file Table_1.docx]

| **Supplementary table 1.** Vitamin contribution to the AIN-93M diet when the recommended vitamin blend AJN-93-VX is fed at 10 g/kg of diet (Adapted from REEVES, 1997). | |
| --- | --- |
| *Mineral* | AIN-93M |
|  | (*mg/kg de dieta*) |
| Calcium | 5000.0 |
| Potassium | 3600.0 |
| Phosphorus | 1992.0 |
| Chloride | 1571.0 |
| Sodium | 1019.0 |
| Magnesium | 507.0 |
| Sulfur - inorganic | 300.0 |
| Iron | 35.0 |
| Zinc | 30.0 |
| Manganese | 10.0 |
| Copper | 6.0 |
| Iodine | 0.20 |
| Molybdenum | 0.15 |
| Selenium | 0.15 |
| *Potentially beneficial mineral element (mg/kg diet)* | |
| Silicon | 5.0 |
| Chromium | 1.0 |
| Fluoride | 1.0 |
| Nickel | 0.5 |
| Boron | 0.5 |
| Lithium | 0.1 |
| Vanadium | 0.1 |
