# Supplementary material for: Physical exercise/melatonin interaction in young rats fed a low-protein diet: a behavioral, electrophysiological, and redox balance analysis
Source: Front Aging Neurosci. 2026 Feb 6;18:1740062. doi: 10.3389/fnagi.2026.1740062 (PMC12920428; doi:10.3389/fnagi.2026.1740062)
Supplement: Supplementary file 2 [file Table_2.docx]

| Supplementary table n° 2. Contribution of vitamins to AIN-93M diet when the recommended vitamin mix is fed at 10 g/kg of the diet (Adapted from REEVES, 1997). | |
| --- | --- |
| **AIN-93M** | |
| *Vitamin (U/kg diet)* | |
| Nicotinic acid (mg) | 30.0 |
| Pantothenate (mg) | 15.0 |
| Pyridoxine (mg) | 6.0 |
| Riboflavin (mg) | 6.0 |
| Thiamin (mg) | 5.0 |
| Folic acid (mg) | 2.0 |
| D-Biotin (mg) | 0.2 |
| Vitamin B12 (µg) | 25.0 |
| Vitamin K (µg) | 750.0 |
| Vitamin A (IU) | 4000.0 |
| Vitamin D (IU) | 1000.0 |
| Vitamin E (IU) | 75.0 |
